# Ambient particle characteristics by single particle aerosol mass spectrometry at a coastal site in Hong Kong: a case study affected by the sea-land breeze

**DOI:** 10.7717/peerj.14116

**Published:** 2022-10-28

**Authors:** Nana Wang, Yanjing Zhang, Lei Li, Houwen Wang, Yunhui Zhao, Guanru Wu, Mei Li, Zhen Zhou, Xinfeng Wang, Jian Zhen Yu, Yang Zhou

**Affiliations:** 1 College of Oceanic and Atmospheric Sciences, Ocean University of Qingdao, Qingdao, China; 2 Institute of Atmospheric Environment Safety and Pollution Control, Jinan University, Guangdong, China; 3 Environment Research Institute, Shandong University, Qingdao, China; 4 Division of Environment, Hong Kong University of Science and Technology, Kowloon, Hong Kong; 5 Department of Chemistry, Hong Kong University of Science and Technology, Kowloon, Hong Kong

**Keywords:** Single-particle, Sea-land breeze, Regional transportation, Air quality, Coastal site

## Abstract

The sea-land breeze (SLB) circulation plays a vital role in the transport of atmospheric pollutants in coastal cities. In this study, a single particle aerosol mass spectrometer (SPAMS) and combined bulk aerosol instruments were deployed to investigate the ambient particle characteristic at a suburban coastal site in Hong Kong from February 22 to March 10, 2013. Significant SLB circulations were captured from March 6–10, 2013, during the campaign. During the SLB periods, air quality worsened, with PM_2.5_ concentrations reaching a peak of 55.6 μg m^−3^ and an average value of 42.8 ± 4.5 μg m^−3^. A total of 235,894 particles were measured during the SLB stage. Eight major sources were identified by investigating the mixing states of the total particles, including the coal-burning related particles (48.1%), biomass burning particles (6.7%), vehicle emission-related particles (16.4%), sea salt (9.2%), ship emission particles (2.7%), dust/steeling industries (3.7%), waste incineration (6.3%), and road dust (3.9%). It was noteworthy that the PM_2.5_ concentrations and particle numbers increased sharply during the transition of land wind to the sea breeze. Meanwhile, the continental sourced pollutants recirculated back to land resulting in a cumulative increase in pollutants. Both individual and bulk measurements support the above results, with high contributions from coal burning, biomass burning, bulk K^+^, and NO_3_^−^, which were probably from the regional transportation from the nearby area. In contrast, the ship and vehicle emissions increased during the SLB period, with a high sulfate concentration partially originating from the ship emission. In this study, field evidence of continental-source pollutants backflow to land with the evolution of sea breeze was observed and helped our current understanding of the effect of SLB on air quality in the coastal city.

## Introduction

Sea-land breeze (SLB) is a local circulation that occurs in coastal areas with thermal differences between sea and land. Sea breeze usually develops toward the coastline as the land surface heats up during the daytime (Crosman & Horel, 2010; Huang et al., 2016). Conversely, land wind typically evolves departing the coastline as the land surface cools down during the nighttime (Arritt, 1993; Hu & Xue, 2016). The direction of SLB makes a clockwise rotation over the diurnal cycle due to the Coriolis forces (Haurwitz, 1947; Moisseeva & Steyn, 2014), which is a simplified method of determining the SLB circulation (Furberg, Steyn & Baldi, 2002; Neumann, 1977). SLB circulation plays a vital role in the transport and diffusion of pollutants in coastal cities (Asimakopoulos et al., 1992; Han et al., 2019; Huang et al., 2016). Land wind prevailing at night can transport pollutants to the coastal areas (Choi, Zhang & Takahashi, 2004; Zhao et al., 2022). Conversely, the dilution of clean sea breeze with a large wind speed can continuously reduce pollutants (Arrillaga et al., 2016; Papanastasiou & Melas, 2009).

Hong Kong is a typical coastal city located in the south of China and adjacent to the South China Sea, which is an important receptor site for anthropogenic pollutants from East Asia. Surrounded by the sea on three sides, Hong Kong has a very intricate coastline. SLB circulation is a common weather phenomenon in Hong Kong. Previous studies have reported that SLB occurs for about 90 days or more each year (Chen et al., 2009). Several numerical simulations have investigated the impact of SLB on the accumulation of pollutants in Hong Kong (Fan et al., 2008; Fan et al., 2011; Liu et al., 2022; Wei et al., 2016). A numerical simulation in Hong Kong found that due to the effect of hilly topography, the thermal effect of the terrain is dominant during the daytime, which enhances the strength of the sea breeze, while the dynamical blocking effect is more pronounced at night, which weakens the strength of the land wind (Chen et al., 2009). Another modeling result in Hong Kong concluded that the SLB can trap air pollutants, resulting in persistent impacts on the air quality (Liu & Chan, 2002b). Moreover, previous studies indicated that the air pollutants concentrations rose due to the convergence zones caused by northward sinking airflow and southward sea breeze (Ma et al., 2022; Wu et al., 2013). Other studies found that during the transition of land wind to the sea breeze, pollutants initially carried to the sea by the land wind may be brought back to land by the redeveloping sea breeze (Igel, Heever & Johnson, 2018; Miao et al., 2015). In this case, pollutants discharged into the upper sea breeze circulation may return to land with the lower sea breeze, resulting in a cumulative increase in pollutant concentrations as a result of the circulation. A field campaign focusing on the effects of the SLB on coastal ozone pollution also observed that pollutants were back to the land during the shift of land wind to sea breeze (Zhao et al., 2022). Ma et al. (2022) used the hourly pollutant concentration data and meteorological data from 2001 to 2018 in Hong Kong to predict daily O_3_ by machine learning models and found that the development of land-sea breeze circulation can effectively trap pollutants, which suggests that the SLB effect is a critical factor influencing the coastal air quality.

However, few researchers analyzed the evolution of aerosol chemical composition affected by the SLB circulation from the field observation perspective, especially using individual particle measurement. Single particle aerosol mass spectrometry (SPAMS) can be used to monitor the size and composition mixing state of individual aerosol particles in real-time, which is beneficial in monitoring trends and capturing the details of transient changes in the various pollution sources (Prather, Hatch & Grassian, 2008). Compared to the traditional bulk filter measurements (Chow et al., 2022; Liu, Baumgartner & Schauer, 2019), the SPAMS can distinguish more specific sources (*e.g*., heavy metals, special organics) of fine particulate matter (Zheng et al., 2014) and the mixing state can supply more direct evidence than the mathematic source apportionment protocol (*e.g*., Positive Matrix Factorization) (Chow et al., 2022; Paatero & Tapper, 1994). In this study, ambient particles at a coastal site in Hong Kong were observed *via* SPAMS to investigate the characteristics of particles from February 22 to March 10, 2013. Significant SLB circulations were captured in the latter days (March 6–10, 2013) of the observation campaign. Meteorological conditions, chemical composition, and diurnal variation observed by single particle measurements were analyzed during the SLB stage.

## Materials and Methods

### Sampling

**Figure 1:**
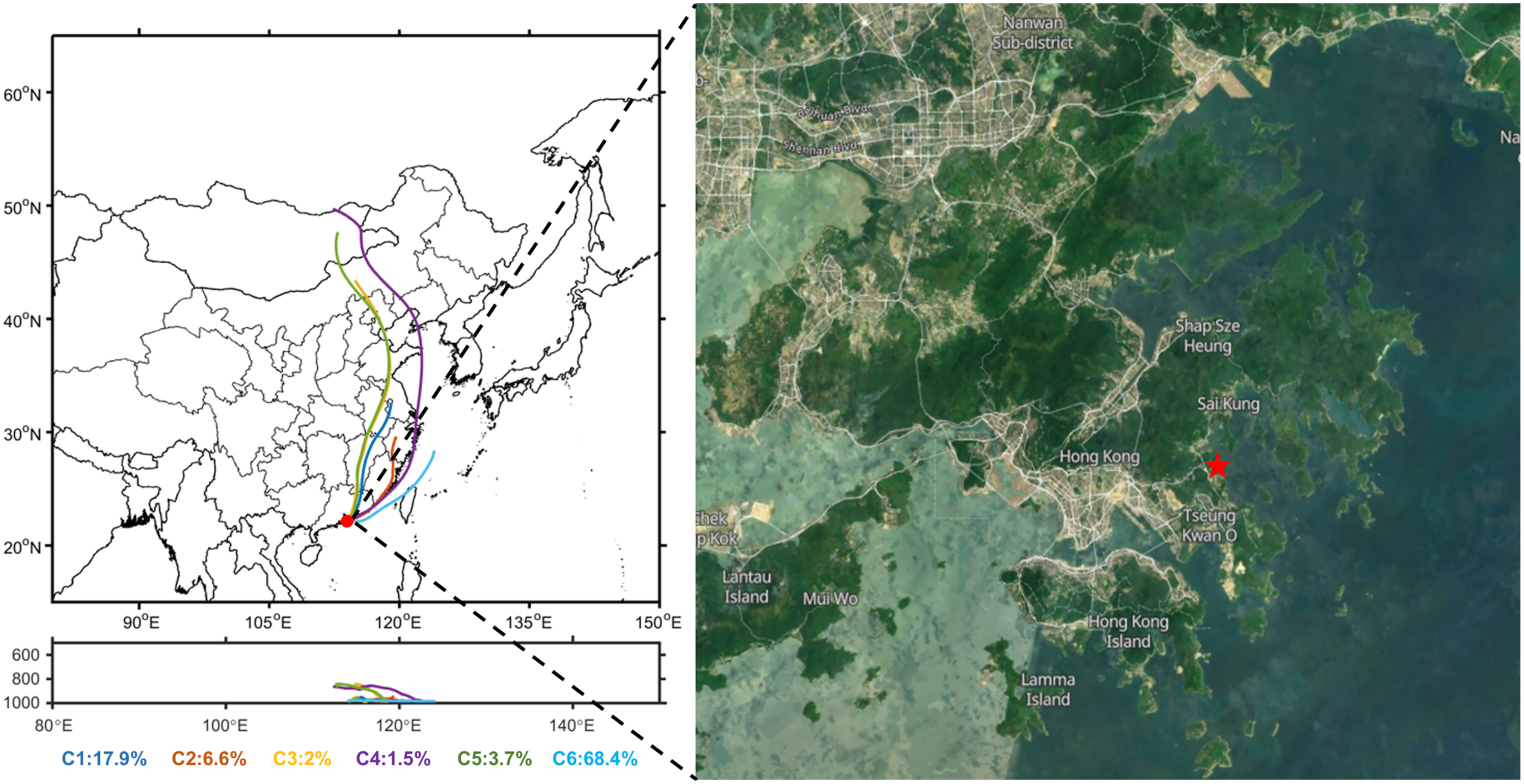
Cluster diagram of the average hourly backward air-mass trajectories during sampling time (February 22 to March 10, 2013) and the location of the observation site.

This campaign was conducted at the Hong Kong University of Science and Technology Air Quality Research Supersite (HKUST AQRS, 22°20′N, 114°16′E, as shown in Fig. 1). The AQRS is located on the rooftop of a building (~20 m high) and has been described in other studies (Griffith et al., 2015; Huang et al., 2014; Wang et al., 2012b; Zhou et al., 2015). The AQRS is a clean suburban coastal receptor site with few residential and commercial areas surrounding it, and the variability of particulate matter is influenced mainly by the environment of the ocean and surrounding areas. Both individual particle and bulk measurements were tracked from February 22 to March 10, 2013.

### Instruments

A single particle aerosol mass spectrometer (SPAMS, Hexin Analytical Instrument Co., Ltd., China) was deployed to analyze a diameter size from 0.2 to 2.0 μm (Li et al., 2011; Li et al., 2014; Zhou et al., 2015). Briefly, particles are introduced into the vacuum through the inlet orifice (~100 μm), where they are focused onto the axis and accelerate when exiting the Aerodynamic Lens. The particle velocity is then calculated by passing the particle through two parallel 532 nm laser beams in the particle size detection zone. The diameter of the individual particle can be calculated according to the particle velocity. The particles then enter the mass spectrometer and are ionized into ion fragments by a pulsed 266 nm Nd: YAG laser. The time-of-flight mass spectrometer can detect the mass-to-charge ratio (m/z) of the positive and negative ions by identifying the mass spectral peaks to get information on the chemical composition and mixing state. This study focused on the particles with both positive and negative signals and named the total particles.

The YAADA2.1 toolkit (http://www.yaada.org/), based on MATLAB, was used to search particular mass spectral features and classify particles. The individual particle data were averaged into an hourly time resolution. During the whole sampling campaign, a total of 790,305 particles were classified by the Art-2a clustering algorithm, with a learning rate of 0.05 and a vigilance factor of 0.7. Generally, 12 major groups of particles were classified (see Table S1 and Fig. S1, detailed mass spectra information was discussed in “Chemical characteristics of single particles during the SLB stage”), accounting for 98% of the total particle number.

Hourly PM_2.5_ concentration was measured by an online instrument (SHARP 5030 Monitor; Thermo Scientific Inc., Franklin, MA, USA) (He et al., 2011). Water-soluble aerosol ions (K^+^, NH_4_^+^, SO_4_^2−^, NO_3_^−^) were measured by MARGA (Metrohm Applikon B.V., Schiedam, The Netherlands) instrument (Trebs et al., 2004; Zhou et al., 2016). All the data were hourly averaged to track the individual particle data.

### Meteorological and backward trajectory analysis

The ERA5 reanalysis dataset (Hersbach et al., 2020) downloaded from ECMWF (https://cds.climate.copernicus.eu/#!/home) is used to investigate the occurrence of sea-land breezes, such as 2-m surface temperature and 10-m wind. Meteorological parameters were also monitored at the site, including surface temperature (Temp), relative humidity (RH), pressure (Pres), surface wind speed (WS), and wind direction (WD).

The HYSPLIT4 model supplied by the NOAA (https://www.arl.noaa.gov/hysplit/) was used to investigate the potential origins of air masses. A total of 72-h backward trajectories for altitude above ground level 300 m (Su et al., 2015; Zhou et al., 2015) were calculated for the whole sampling period (February 22 to March 10, 2013). As shown in Fig. 1, the air masses were grouped into six clusters, with the clusters of C1–C5 being continental air masses, while C6 being the marine air mass. The SLB stage was mainly influenced by the C6 air masses. Moist and clean marine air masses from the East China Sea passing through the Taiwan Strait dominated during this stage.

## Results

### General statistics of meteorological parameters and pollution conditions

**Figure 2:**
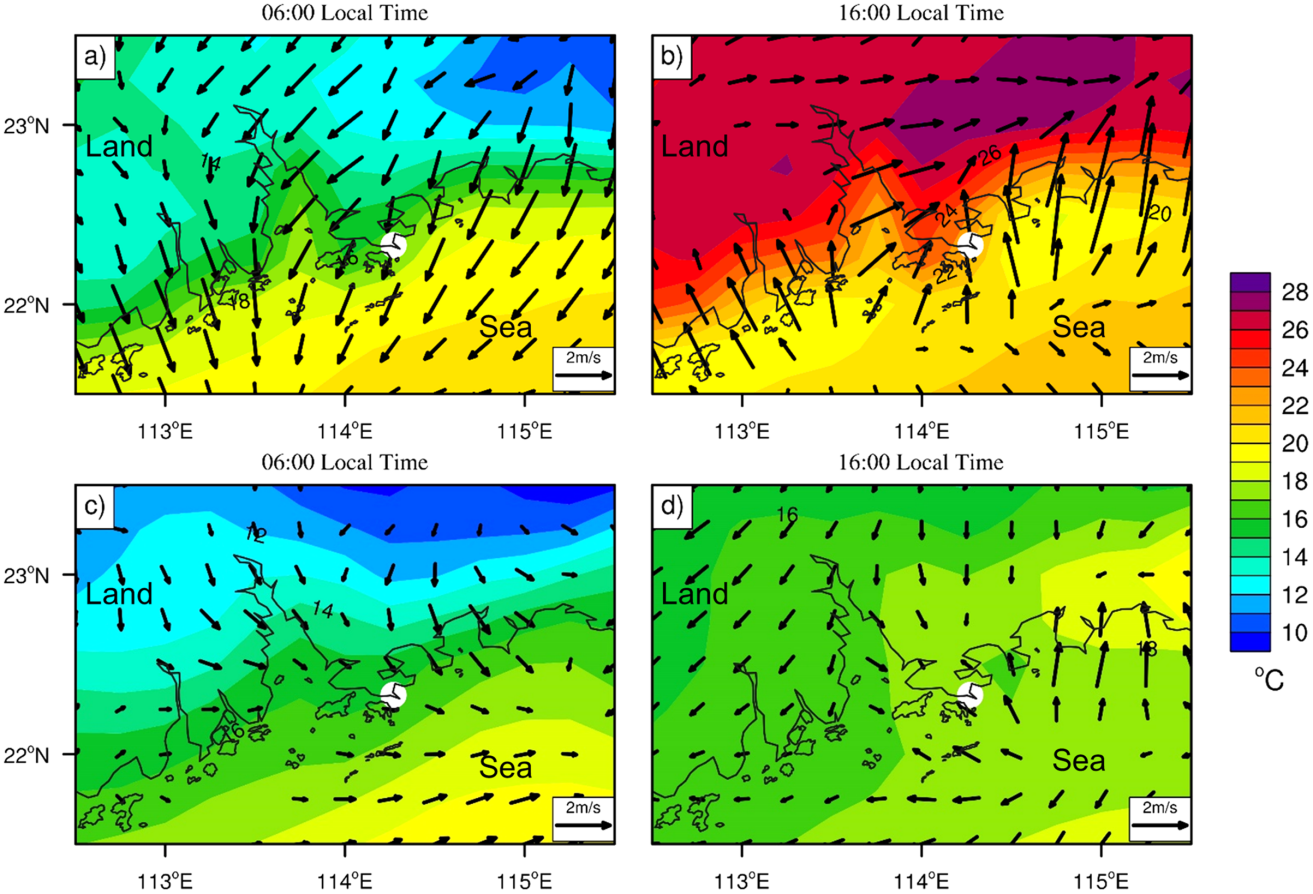
Hourly average of 2-m surface temperature (shading, units: °C) and anomalous (Local Time minus daily mean) of 10-m wind vector (arrow, units: m/s). At (A) 06:00 local time, (B) 16:00 local time during the SLB stage, and (C) 06:00 local time, (D) 16:00 local time during the non-SLB stage. The white dots represent the sampling site.

The time trends of meteorological parameters and pollutants concentrations during the whole sampling period are shown in Figs. S2 and S3. As the *in-situ* site data shown in Fig. S2A, during the period from March 6 to March 10, the wind exhibited typical SLB characteristics with clockwise rotation of wind direction over the diurnal cycle. To better identify the sea-land breeze, the ERA5 reanalysis dataset was processed by removing the background wind component from the local time wind field by deducting the daily mean (Jin et al., 2022). Through this identification method, we defined the period of March 6–10, 2013 as the SLB stage and the period from February 22 to March 5, 2013 as the non-SLB stage. Hourly average of 2-m surface temperature and anomalous of 10-m wind vector are shown in Fig. 2. The land surface is cooler during the nighttime than the ocean, with the northerly winds predominating when the offshore wind is well developed over Hong Kong (Fig. 2A, 06:00 local time). As the offshore wind developed, it transformed into onshore wind due to the land surface warming after sunrise, with a larger wind speed and a persistent southeasterly wind at 16:00 local time, as shown in Fig. 2B. A significant wind direction shift was exhibited from northerly to southwesterly during the daily cycle, which is in accordance with previous field observation (Moisseeva & Steyn, 2014) and numerical experiments (Lu et al., 2009) during the SLB circulation. The wind field during the non-SLB stage (Figs. 2C, 2D) showed that background wind dominated with very slight southeast wind from the sea, while during the SLB stage strong sea breeze (over 2 m/s) dominated due to the large temperature differences between land and sea.

Table 1 compared the general information during the SLB period with the non-SLB period. SLB stage showed a similar average temperature (18.7 ± 3.0 °C) and pressure (101,623 ± 293.2 Pa) with the Non-SLB period, but less RH (69.9 ± 14.8%) and wind speed (0.8 ± 0.9 m/s). During the SLB stage, PM_2.5_ concentrations reached a maximum of 55.6 μg m^−3^, with an average value (42.8 ± 4.5 μg m^−3^) exceeding the non-SLB period (28.9 ± 10.6 μg m^−3^) by 48.1%. The hourly particle number averaged 2,432 h^−1^, during the SLB stage, which was 14.9% higher than the non-SLB period. Air quality worsened during the SLB stage significantly.

**Table 1:** Comparison of meteorological parameters and pollutants on average during the SLB stage *vs* the non-SLB stage.

Atmospheric parameters	SLB stage (March 6–10, 2013)	Non-SLB stage (February 22 to March 5, 2013)
Temp (°C)	18.7 ± 3.0	17.9 ± 2.6
RH (%)	69.9 ± 14.8	80.1 ± 15.7
Pres (Pa)	101,623 ± 293.2	101,740 ± 424.6
WS (m/s)	0.8 ± 0.9	1.3 ± 1.0
PM_2.5_ concentrations (μg m^−3^)	42.8 ± 4.5	28.9 ± 10.6
Total particle number	235,894	554,411
Hourly particle number (hour^−1^)	2,432	2,116
Major backward air-mass trajectories sources	Marine air mass	Continental and marine air mass

### Chemical characteristics of single particles during the SLB stage

**Figure 3:**
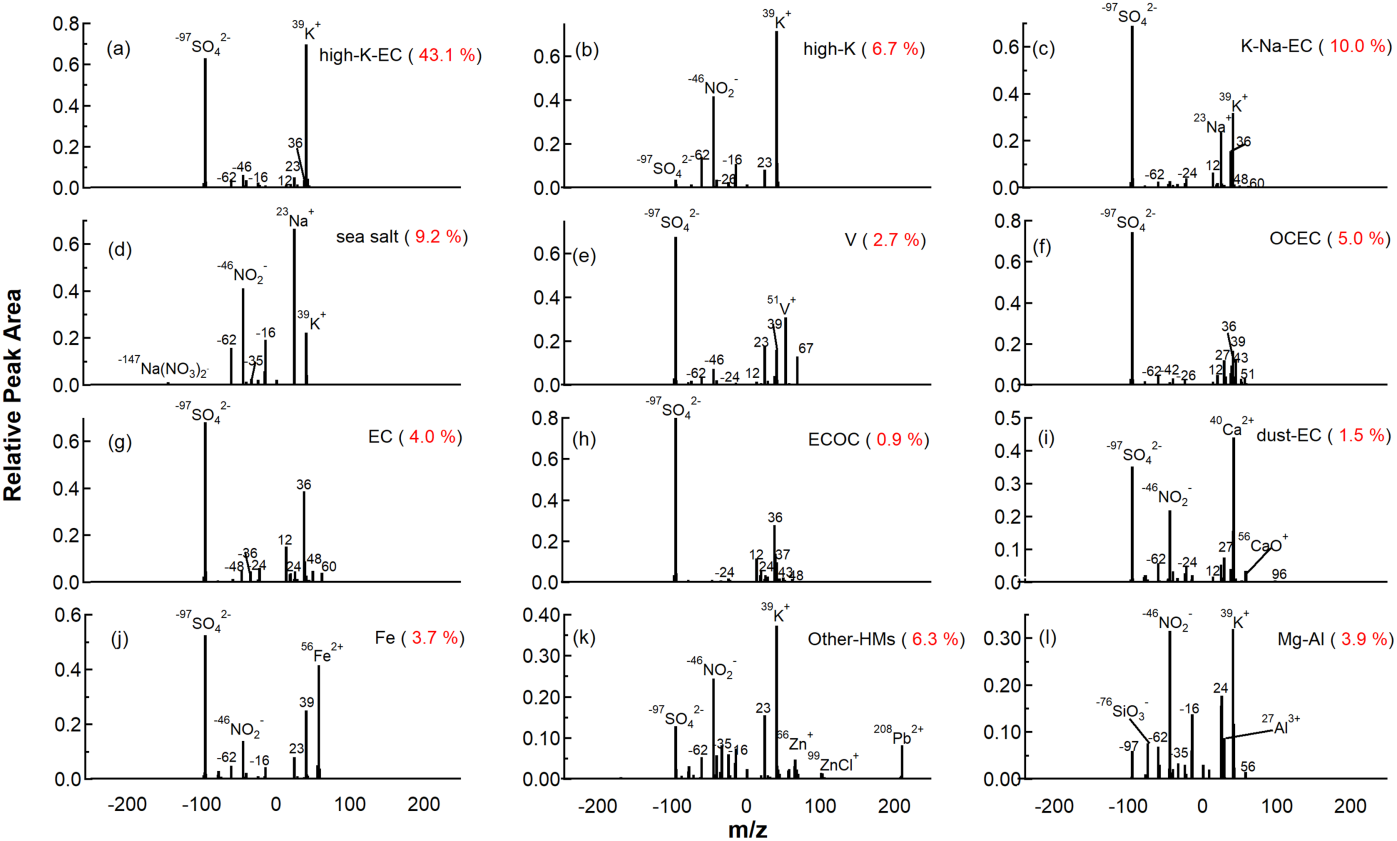
Average mass spectra of 12 major particle types classified using the Art-2a clustering algorithm. The red number in the brackets is the fraction contribution to the total particle count.

**Figure 4:**
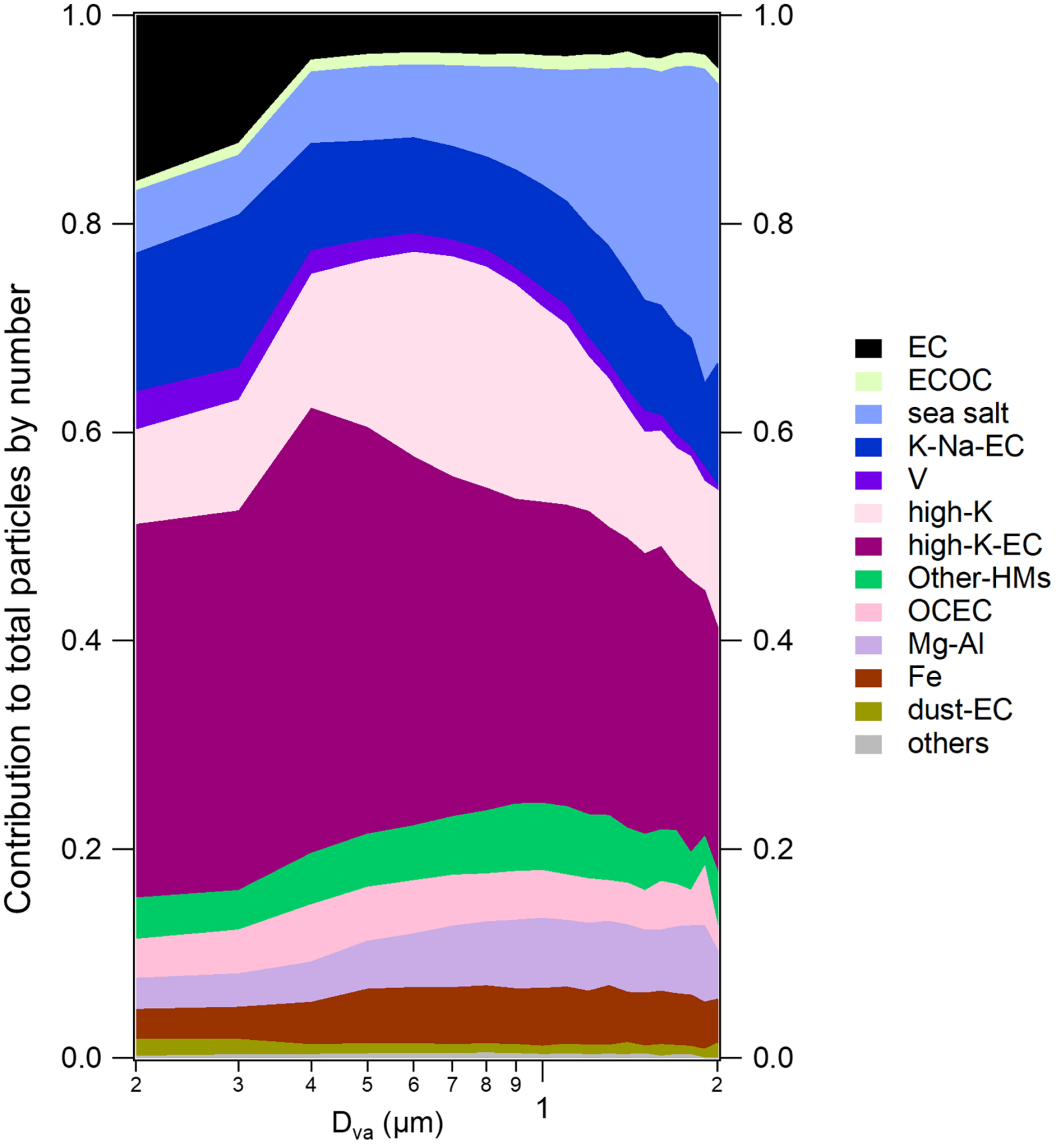
Size-resolved number fractions of classified groups to the particles during the SLB stage.

A total of 235,894 particles were measured during the SLB stage (March 6–10, 2013). These particles were classified into 12 types and further grouped into five major classes according to mass spectral similarity, time trends, and particle size distribution; other particles were grouped as undefined particles. The average spectrum of each group and normalized size distribution were shown in Figs. 3 and 4, respectively. The zoom-in figures of the average spectrum can refer to Fig. S4.

#### K-rich

Three types of K-rich particles were identified as high-K-EC, high-K, and K-Na-EC. High-K-EC particles were the most abundant, accounting for 43.1% of the total particles. The average ion mass spectra of high-K-EC particles are shown in Fig. 3A, which were featured by strong signals at ^39^K^+^, along with less intense carbonaceous ion peaks at ^12^C^+^ and ^36^C_3_^+^ in the positive spectra. The negative spectra of high-K-EC exhibited intense sulfate signals at ^97^HSO_4_^−^, together with less intense nitrate peaks at ^46^NO_2_^−^, ^62^NO_3_^−^, and the weak carbonaceous ion peaks at ^24^C_2_^−^, ^36^C_3_^−^ and ^48^C_4_^−^. Figure S4A shows the detail of the minor spectra peak of ^208^Pb^+^. The high intensity of element carbon and sulfate and the presence of ^208^Pb^+^ indicate that the high-K-EC particles might be from coal burning (Guazzotti et al., 2003; Wang et al., 2016; Xu et al., 2017; Zhao et al., 2017).

High-K particles accounted for 6.7% of the total number of particles. As shown in Fig. 3B, the positive spectra were characterized by strong signals at ^39^K^+^, together with other weak ion peaks representing ^23^Na^+^, ^56^Fe^+^, ^64^Zn^+^, and ^208^Pb^+^. Unlike high-K-EC particles, the negative spectra of high-K group existed with strong nitrate signals at ^46^NO_2_^−^ and ^62^NO_3_^−^, and some organic nitrate signals at ^26^CN^−^, suggesting they may not come from coal burning but from biomass burning (Silva & Prather, 2000; Zauscher et al., 2013). A considerable fraction of high-K particles were mixed with ^35^Cl^−^ (33.7%), and 35.6% of high-K particles contained levoglucosan ion fragments (^45^CHO_2_^−^ and ^59^C_2_H_3_O_2_^−^) (Fig. S4). The chloride and levoglucosan are considered tracers of fresh biomass burning because they degrade over time (Bi et al., 2011; Chen et al., 2017). The strong nitrate signals at ^46^NO_2_^−^ and ^62^NO_3_^−^ implied that the particles have undergone the aging process (Liu, Wenzel & Prather, 2003; Pratt et al., 2011). Thus, the high-K particles may come from a mixed source of fresh and aged biomass burning.

K-Na-EC particles contributed 10% of the total particles. As shown in Fig. 3C, K-Na-EC particles exhibited typical elemental carbon ion clusters (^12^C^+/−^, ^24^C_2_^+/−^, ^36^C_3_^+/−^, ^48^C_4_^+/−^, and ^60^C_5_^+/−^), with ^36^C_3_^+^ as the dominant fragment. The positive spectrum of the K-Na-EC particles featured peaks at ^39^K^+^ and ^23^Na^+^. In the negative spectra, an intense sulfate peak at ^97^HSO_4_^−^ and weak nitrate signals at ^46^NO_2_^−^ and ^62^NO_3_^−^ existed, which were similar to the previous studies (Li et al., 2013; Sodeman, Toner & Prather, 2005). Sodium is mainly from marine or combustion sources (Yan et al., 2018). The temporal variation of the fraction of K-Na-EC particles showed considerable correlations with EC (r^2^ = 0.67) and ECOC particles (r^2^ = 0.41) (Fig. S5), indicating that the K-Na-EC particles may come from similar vehicle emissions (Li et al., 2014; Zhao et al., 2017).

#### Marine-sourced species

The number fraction of sea salt particles during the SLB stage is 9.2%. As shown in Fig. 3D, the positive spectra exhibited intense peaks of ^23^Na^+^ and less intense peaks of ^39^K^+^, ^46^Na_2_^+^, ^62^Na_2_O^+^ and ^81^Na_2_Cl^+^ (see Fig. S4D). In the negative spectra, strong nitrate signals at ^46^NO_2_^−^ and ^62^NO_3_^−^, along with weak signals at ^35 37^Cl^−^, also the signal of ^147^Na(NO_3_)_2_^−^, implying the aging process by reacting with HNO_3_ to release hydrogen chloride to the gas phase and form nitrate (Braun et al., 2017; Dall’Osto & Harrison, 2006), which is called chloride depletion in the sea salt particles (Ault et al., 2014; Bondy et al., 2017; Su et al., 2021). Most sea salt particles were in coarse mode (Fig. 4) and were the largest among the 12 types of particles.

Vanadium (V) particles contributed 2.7% to the total particles by number. Ship emission particles were composed of V particles since vanadium is associated with heavy fuel oil combustion (Ault et al., 2010; Moldanová et al., 2009; Zhou et al., 2022). As shown in Fig. 3E, Vanadium peaks at ^51^V^+^ and ^67^VO^+^ are the dominant peaks in the positive spectra of V particles. Meanwhile, elemental carbon ion clusters (such as ^12^C^+^, ^24^C_2_^−^ and ^36^C_3_^+/−^) and the intense sulfate peaks at ^97^HSO_4_^−^ reflected that the V particles were freshly emitted (Healy et al., 2009; Yan et al., 2018). Previous studies have found that fresh ship emission particles produced very low nitrate signals in the mass spectra, as commonly observed in combustion-source characterizations, probably due to the high sulfur content in the residual oil (Wang et al., 2019).

#### Carbonaceous species

Carbonaceous species accounted for 11.5% of the total number of particles classified chemically, which were divided into four major subgroups: organic carbon (OCEC), elemental carbon (EC), mixed ECOC, and dust-EC. The OCEC particles featured with typical organic carbon ion clusters (such as ^27^C_2_H_3_^+^, ^37^C_3_H^+^, ^43^C_2_H_3_O^+^ and ^51^C_4_H_3_^+^) with a few elemental carbon ion clusters (^12^C^+^ and ^36^C_3_^+^) in Fig. 3I. The negative spectra of OCEC particles featured strong sulfate signals at ^97^HSO_4_^−^ and weak nitrate signals at ^46^NO_2_^−^ and ^62^NO_3_^−^. OCEC particles mainly arise from the combustion source (Bi et al., 2011; Oduber et al., 2021). The temporal variation of OCEC particles showed good correlations (r^2^ = 0.50) with high-K-EC particles (Fig. S6), suggesting that coal burning may be a major source of OCEC particles.

The EC particles exhibited typical elemental carbon ion clusters (^12^C^+^, ^24^C_2_^+/−^, ^36^C_3_^+/−^, ^48^C_4_^+/−^, and ^60^C_5_^+/−^) in Fig. 3H, with ^36^C_3_^+^ as the dominant fragment, which was similar to K-Na-EC. In the negative spectra, an intense sulfate peak at ^97^HSO_4_^-^ was exhibited. As shown in Fig. 4, 62.3% of EC particles were below 400 nm and from the primary combustion sources. Previous studies reported that EC was a typical tracer of vehicle emissions (Sodeman, Toner & Prather, 2005; Yang et al., 2017). As the diurnal variation of EC particles exhibited peaks at rush hours (see Fig. S7), we speculate that EC particles were mainly from fossil fuel combustion by local emission.

As shown in Fig. 3J, the ECOC particles exhibited typical elemental carbon ion clusters (such as ^12^C^+^, ^24^C_2_^+/−^, ^36^C_3_^+/−^, ^48^C_4_^+/−^ and ^60^C_5_^+/−^) with few organic carbon ion clusters (^37^C_3_H^+^ and ^39^C_3_H_3_^+^), which was in contrast to the OCEC particles. In the negative spectra, an intense sulfate peak at ^97^HSO_4_^−^ was exhibited, which was similar to the EC particles. The ECOC particles showed a similar trend to the EC particles (see Fig. S3), indicating that the ECOC may be the oxidation of EC particles and come from the same source (Dall’Osto & Harrison, 2006).

The dust-EC was characterized by calcium signals at ^40^Ca^+^, and its oxide adduct ions at ^56^CaO^+^ and ^96^Ca_2_O^+^. Minor elemental carbon signals at ^12^C^+^ and ^24^C_2_^−^ were also presented in Fig. 3K, suggesting that they might arise from a combustion source. There was a ^27^Al^+^ peak at the positive spectra of dust-EC particles, which was similar to the results obtained by previous studies (Li et al., 2014). The negative spectra of dust-EC particles featured strong sulfate signals at ^97^HSO_4_^−^ and minor nitrate signals at ^46^NO_2_^−^ and ^62^NO_3_^−^. Calcium was a typical detergent additive used in vehicular lubricants to neutralize acidic combustion by-products, including calcium carbonate and calcium sulfonate (Baderna et al., 2012; Shields, Suess & Prather, 2007). This suggests that the dust-EC particles were from fossil fuel combustion by local emission and EC particles. As shown in Fig. 4, the size distribution also suggests a small particle size of this group, supporting its combustion source rather than dust.

#### Metal

The positive spectrum of Fe particles featured intense peaks at ^54/56^Fe^+^ and less intense peaks at ^39^K^+^ and ^23^Na^+^ in Fig. 3F. The negative spectra featured strong sulfate signals at ^97^HSO_4_^−^ and weak nitrate signals at ^46^NO_2_^−^ and ^62^NO_3_^−^, similar to previous studies of iron-containing particles from industry emissions (Lin et al., 2019; Zhou et al., 2020). Fe particles were also closely related to the air mass from North China (see Fig. S2. C3 and C5 were the clusters of long-range transportation in Fig. 1). Thus, we supposed that Fe particles were mainly from the long-range transportation of dust/steeling industries. Some peaks also occurred with the influence of marine air masses (C6 in Fig. S2), and a detailed discussion can be found in “Diurnal variations of the meteorological parameters and the chemical compositions of single particles”.

As shown in Fig. 3G, heavy metal tracers presented in the positive spectra of Other-HMs particles, including ^208^Pb^+^, ^64/66/68^Zn^+^, and ^99/101/103^ZnCl^+^. The negative spectra marked nitrate signals at ^46^NO_2_^−^ and ^62^NO_3_^−^, along with ^97^HSO_4_^−^. Also, the chloride signals of ^35^Cl^−^, organic nitrogen signals of ^26^CN^−^ and ^42^CNO^−^. Lead and zinc chlorides are emitted in the gas phase of high-temperature combustion sources, especially the waste incinerators (Moffet et al., 2008). Due to their relatively low boiling points (950 °C and 732 °C, respectively), these chlorides of heavy metals can condense into the particle phase, forming Cl-contained particles (Hu et al., 2003; Olmez et al., 1988; Ondov & Wexler, 1998). Thus we perceived that the Other-HMs particles might be from waste incineration.

#### Fireworks/dust

Mg-Al particles made up 3.9% of total particles. As shown in Fig. 3L, ions at ^24^Mg^+^, ^27^Al^+^, ^56^Fe^+^ and peaks at ^39^K^+^ were exhibited in the positive spectra. The nitrate signals at ^46^NO_2_^−^ and ^62^NO_3_^−^ were strong, while the silicon signature at ^60^SiO_2_^−^ and ^76^SiO_3_^−^, chloride peaks at ^35^Cl^−^ and sulfate signals at ^97^HSO_4_^−^ were less intense relatively in the negative spectra. In the previous studies, similar MgAlSi particles were identified as fireworks ash, which is either related to black powder components or crustal elements (Li et al., 2016), since soil dust is usually added during manufacturing fireworks/crackers could lead to the re-suspension of road dust (Tian et al., 2014). Sr and Ba are typical fireworks tracers used as colorants for fireworks (Liu et al., 1997; McGuire et al., 2011). However, only during the non-SLB stage found a high correlation (r^2^ = 0.65 and 0.69) between Mg-Al particles and fireworks tracers at ^88^Sr^+^, ^138^Ba^+^/^154^BaO^+^ indicates that the Mg-Al particles were from fireworks source (Fig. S8). The sampling period is in the first month of the lunar year in China, and setting off fireworks is a traditional celebration, especially in February. During the SLB stage, the worse correlation (r^2^ = 0.01 and 0.35) between Mg-Al particles with Sr and Ba may indicate the dust source of these Mg-Al particles (Li et al., 2014). According to the discussion of diurnal variation in “Combined the bulk and single particle analysis to understand the effects of SLB on the PM_2.5_ pollution”, the Mg-Al particles during the SLB period were mainly related to road dust.

## Discussion

### Overview of the sources of single particles during the SLB stage

The classified groups and potential sources of the corresponding particles during the SLB stage were summarized in Table 2. Eight sources were identified after the detailed analysis based on the chemical mixing, the size distribution, and the temporal variations. Coal burning related particles, including high-K-EC and OCEC, were the most abundant source, accounting for 48.1% of the total particles. The fraction of coal burning related particles showed a significant increase during the SLB stage compared with the entire period (39.2%, Table S1). During the SLB stage, the hourly particle numbers of high-K-EC and OCEC particles increased by 40.3% and 17.0%, respectively, when compared with those over the entire sampling campaign (Table S1).

**Table 2:** Summary of the different groups classified by Art-2a during the SLB stage.

	Groups	Hourly number	Fraction in total (%)	Source
K-rich	High-K-EC	1,061	43.1	Coal burning
	High-K	164	6.7	Biomass burning
	K-Na-EC	246	10.0	Vehicle emission
Marine-sourced species	Sea salt	225	9.2	Sea salt
	V	68	2.7	Ship emission
Carbonaceous species	OCEC	124	5.0	Coal burning
	EC	99	4.0	Vehicle emission
	ECOC	22	0.9	Vehicle emission
	Dust-EC	38	1.5	Vehicle emission
Metal	Fe	92	3.7	Dust/steeling industries
	Other-HMs	155	6.3	Waste incineration
Fireworks/dust	Mg-Al	95	3.9	Road dust
Undefined		62	3.0	--

Marine source related particles, including sea salt particles and V for ship emission contributed 9.2% and 2.7% to the total particles, with 11.9% in sum. Although the average fraction of marine source particles was slightly higher during the SLB stage compared with the entire campaign (10.1%, Table S1), the hourly particle number of sea salt particles and V particles increased by 23% and 74.4%, respectively (Table S1).

K-Na-EC, EC, ECOC, and dust-EC particles were generally from vehicle emissions, accounting for 16.4% of the total particles. The overall contribution of vehicle emission during the SLB stage was comparable with the whole campaign (15.7% Table S1), suggesting the stable local emission characteristics (Sodeman, Toner & Prather, 2005; Yang et al., 2017).

Dust/steeling industries (Fe) and fireworks/dust (Mg-Al) contributed 3.7% and 3.9% to the total particles, respectively, which is 5.0% and 5.1% for the entire campaign (Table S1). The hourly particle number also decreased by 15.6% and 15.9% during the SLB stage compared with the whole sampling period. These two factors may be related to the transport from mainland China (Moffet et al., 2012; Takahashi et al., 2011), resulting in less contribution under the influence of the marine source air mass. In contrast, both the fraction and hourly particle number of waste incineration increased during the SLB stage (Table S1). We noticed that the waste incineration source particles showed a significant contribution to the total particles when the air masses pass through the coastline or from the sea (C6 and C2, see Fig. S2), probably caused by more municipal solid waste (MSW) incineration located along the coastline (Lu et al., 2017) or in the nearby cities in Shenzhen and Guangzhou (Tang et al., 2018).

In sum, during the SLB period, seven general sources of PM_2.5_ particles were identified as coal burning (48.1%), vehicle emission (16.4%), marine source (11.9%), biomass burning (6.7%), waste incineration (6.3%), dust/steeling industries (3.7%), and road dust (3.9%). Previous studies on source apportionment at coastal sites worldwide have shown that combustion or vehicular traffic is the major source, also the marine source is important due to these sites are close to the sea (Arndt et al., 2017; Healy et al., 2010; Taiwo et al., 2014; Yang et al., 2017). The source apportionment characteristics of these coastal sites vary depending on the particular geographical location and local emission sources.

Source appointment for an urban site located in the Pearl River Delta (PRD) region conducted in winter at Guangzhou by SPAMS showed that coal combustion, vehicle exhaust, and secondary ion were the most abundant particle sources, accounting for 28.5%, 17.8%, and 18.2%, respectively. Other minor emission sources were mainly dust, sea salt, and biomass burning, with the percentage of 12.7%, 2.7%, and 12.5%, respectively (Yang et al., 2017). Compared to the result in Guangzhou, the high contribution of coal burning suggested that our site is more influenced by the transportation of continental pollutants. Marine source particles were higher than in Guangzhou because the site in Hong Kong is a coastal site and is affected more by the sea breeze. Overall, regional transportation played an important role in air quality during the SLB period in this study.

### Diurnal variations of the meteorological parameters and the chemical compositions of single particles

**Figure 5:**
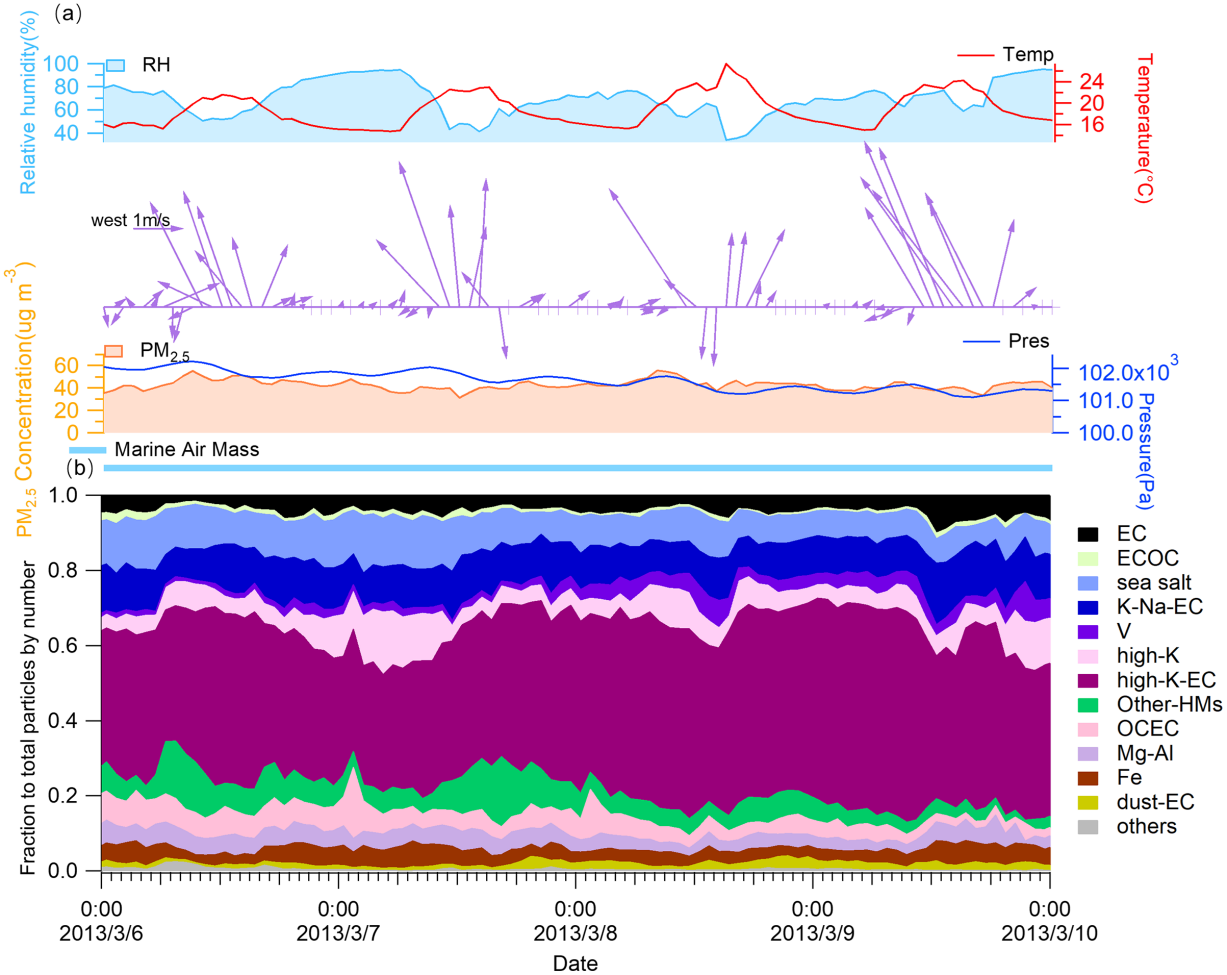
Time trends of (A) meteorological parameters, PM_2.5_ concentrations, and (B) the contribution of each group to the total particles by particle number during the SLB stage.

Figure 5 shows the time series of ambient parameters and percent contributions by individual groups to total particle numbers during the SLB stage. Significant diurnal variations of atmospheric parameters can be seen during the SLB stage. The highest temperature occurred in the early afternoon and the lowest at night. The RH, on the other hand, showed the opposite trend. The synoptic flow followed a similar tendency to the temperature, with the southeasterly breeze reaching the maximum speed during the midday, while the wind was usually at a lower speed or even calm condition during the nighttime. The PM_2.5_ concentrations were persistently high (in the proximity of 40 μg m^−3^), which might result from the impact of the SLB circulation that can trap air pollutants (Liu & Chan, 2002a; Liu & Chan, 2002b).

**Figure 6:**
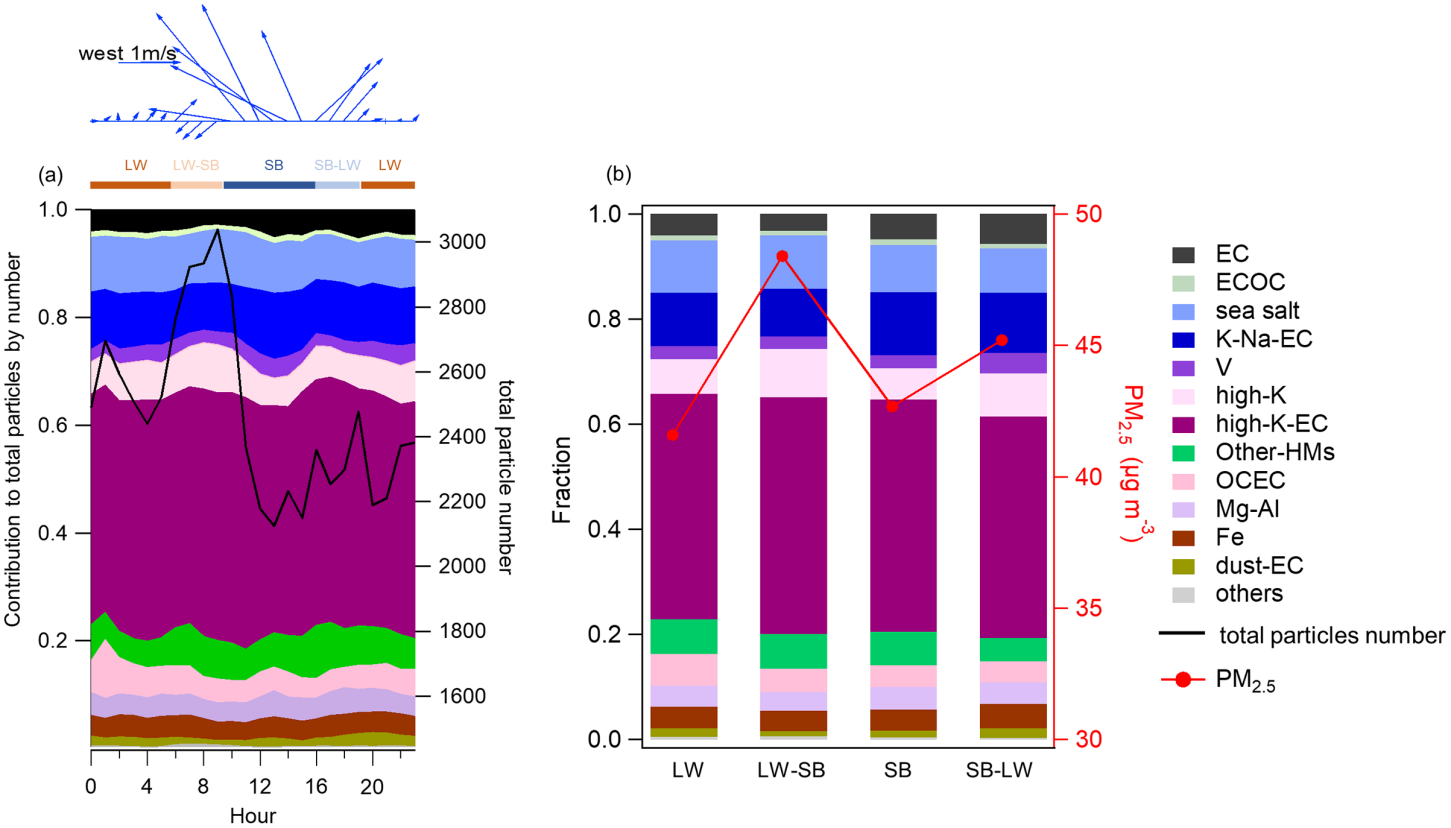
Average diurnal variations and contributions. (A) Average diurnal variations of wind and contribution of each classified group to the total particles by particle number and total particles number during the SLB stage and (B) average of the contribution of each classified group to the total particles by particle number and the concentration of PM_2.5_ during LW, LW-SB, SB, and SB-LW periods.

To further undersatnd the effects of SLB on atmospheric pollutants, we analyzed the specific characteristics of the diurnal variations of wind and PM_2.5_ concentrations with the contribution of each group to the total particles by particle number (Fig. 6).

Based on the orientation of the coastline where the sampling site is located (Fig. 1), we defined onshore and offshore winds by wind direction. Specifically, the weak land wind is typically southwesterly, westerly, and northerly, while the sea breeze usually is southeasterly, easterly and southerly, based on the data measured in the field.

As shown in Fig. 6A, the period from 0:00 to 6:00 is dominated by the land wind (LW). The land wind is characterized by light (0.18 ± 0.14 m/s) or even calm wind speed and southwest wind direction. We had shown the offshore wind at 6:00 in the averaged anomalous fields (Fig. 2A). The wind direction shifted from southwest to northeast with a higher (0.41 ± 0.09 m/s) wind speed from 07:00 to 09:00. This transition period of land wind to sea breeze (SB) is denoted by LW-SB. As the sea breeze evolves, it is well established from 10:00 to 15:00. During this period, the sea breeze is characterized by high wind speed (1.96 ± 0.38 m/s) and a southeasterly direction. From 16:00 to 19:00, the wind direction shifts again, from southeast to southwest, with a decrease in wind speed (0.98 ± 0.53 m/s). We define this period as the transition stage from sea breeze to land wind (SB-LW). A calm synoptic condition was formed with the further development of the land wind (LW).

The average diurnal variations in PM_2.5_ concentrations, particle number, and the fraction of each classified group to the particles by number are illustrated in Fig. 6B. It was noteworthy that the PM_2.5_ concentrations increased significantly during the LW-SB stage with a larger wind speed (0.30–0.53 m/s) than in the LW stage (0.01–0.18 m/s). With the evolution of the sea breeze, the PM_2.5_ concentrations decreased rapidly as a result of the dilution of clean southeastern sea breeze with a large wind speed (1.20–2.36 m/s). Meanwhile, the number of particles showed a similar pattern by the SPAMS analysis (Fig. 6A). In contrast, during the non-SLB period, the PM_2.5_ concentration decreased from 6:00 to 9:00 and then increased as shown in Fig. S9, which was the reverse of the trend during the SLB period. Several types of particles showed a similar increase in contribution during the LW-SB stage, including high-K-EC and high-K groups, which were identified as continental sourced coal burning and biomass burning, respectively. It was notable that the winds came from the sea, yet the ambient had an increased component of particles from continental sources rather than marine sources during the LW-SB stage. This paradox might account for the fast growth of the PM_2.5_ concentrations in the meantime.

### Combined the bulk and single particle analysis to understand the effects of SLB on the PM_2.5_ pollution

**Figure 7:**
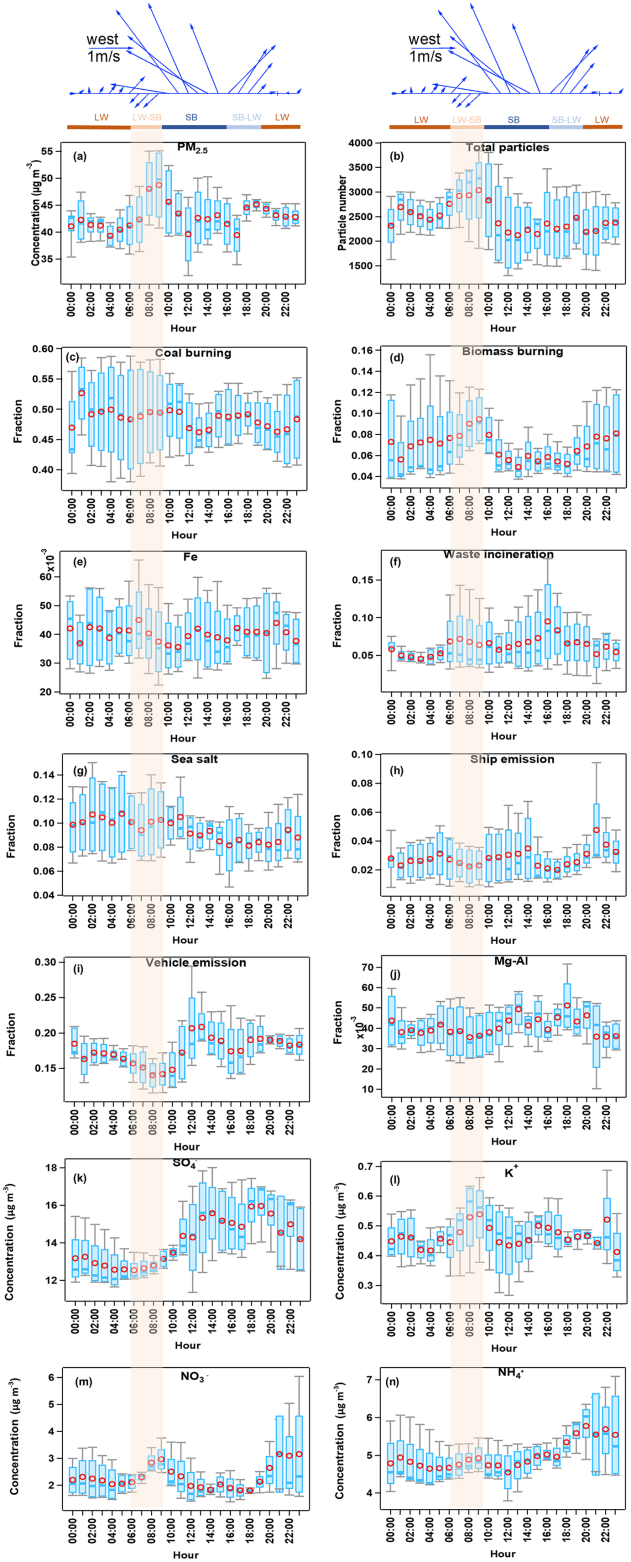
Diurnal variation of wind and species. (A) The concentrations of PM_2.5_ measurements by SHARP, (B) the total particles number, (C–J): contribution of sources (coal burning, biomass burning, Fe, waste incineration, sea salt, ship emission, vehicle emissions, and Mg-Al) to the total particles by number measurements by SPAMS, and (K–N): bulk concentrations of SO_4_^−^, K^+^, NH_4_^+^, and NO_3_^−^ by MARGA (box and whisker denote the 25th and 75th percentiles, and the 5th and 95th percentiles, respectively, and the circle and line in the box represent the mean and the median).

We statistics the diurnal variation of each source of particles and the major bulk ions concentrations in Fig. 7 by Igor toolkits (Wu, Wu & Yu, 2018; Wu & Yu, 2018). A bi-modal diurnal variation was observed for the PM_2.5_ concentrations (Fig. 7A). As discussed in “Diurnal variations of the meteorological parameters and the chemical compositions of single particles”, a significant increase in PM_2.5_ concentrations was observed during the LW-SB transition period, with a northeast wind direction. Previous studies found that during the transition of land wind to the sea breeze, air pollutants, initially carried to the sea by the land wind, may be brought back to the land by the redeveloping sea breeze. The pollutants discharged into the upper sea breeze circulation may return to land with the lower sea breeze, resulting in a cumulative increase in pollutant concentrations (Igel, Heever & Johnson, 2018; Miao et al., 2015). This phenomenon may cause the first PM_2.5_ increase during the LW-SB period, as shown in Fig. 7A. The northwest wind may also bring the pollutants from short-range transport from the nearby cities. Such as, the industrail Fe and waste incineration emissions showed a peak at the beginning of the LW-SB period (peak at 7:00, Figs. 7E and 7F). The coal burning and biomass burning (Figs. 7C and 7D) related particles increased continuously and peaked simultaneously as K^+^, NH_4_^+^ and NO_3_^−^ (9:00 Figs. 7L–7N). As the general air mass during the SLB stage is from the ocean area (Fig. 1), we suspected the high land-based aerosols should be a regional transport from the PRD region or the nearby area. We compared the mixing state of particles during the LW-SB period and the super-long range transport of air masses during C3 and C5 (Fig. 1) in Fig. S10. Relatively higher contributions of particles from coal burning (49.5%) but less biomass burning (9.2%) were observed in the LW-SB period compared with that in the super-long range transport period (43.0% and 13.8% for coal burning and biomass burning, respectively). This information indicated that the high coal burning related particle during the LW-SB period might be significantly influenced by the transportation from regional PRD or the nearby area. While the biomass burning aerosols are more abundant during the super-long range transport.

After then with the well-developed SB, continental aerosols from biomass burning and waste incineration source decreased, while the marine source factors (sea salt and ship emission) kept relatively high or stable contributions to the total particles (Figs. 7G and 7H). In addition, the vehicle emission showed significant contributions around 12:00 and 13:00 (Fig. 7I), consistent with the rush hour with heavy traffic at HKUST. Fe and Mg-Al with a similar peak around 13:00 may suggest a crustal dust source of these two groups produced together with the vehicle activities during this period. The bulk SO_4_^2−^ continued to increase and peaked around 14:00 (Fig. 7K), when the air mass changed from the SB to LW. The SO_4_^2−^ showed a similar variation with the ship emission. Generally, the sulfates are primarily produced through the gas-phase oxidation of SO_2_ by the OH radical followed by nucleation and condensational growth, or secondarily produced by the heterogeneous uptake of SO_2_ on pre-existing particles or through the photochemistry pathway followed by being oxidized (Bie et al., 2021; Liu et al., 2020; Seinfeld, Pandis & Noone, 1997; Wang et al., 2012a). Ships emit large amounts of SO_2_ which are associated with heavy fuel oil combustion (Ault et al., 2010; Moldanová et al., 2009; Zhou et al., 2022). Therefore, we supposed that partial sulfate was attributed from the ship emission. A second small PM_2.5_ peak existed around 18:00 during the SB-LW transition period and kept general stable concentrations during the LW period. The particles from continental sources and nitrate were back to high concentrations under the influence of LW.

## Conclusions

In this study, a SPAMS was deployed to identify the ambient particle characteristic at a coastal site in Hong Kong from February 22 to March 10, 2013. Salient SLB circulations were captured in the latter days (March 6–10, 2013) of the observation campaign. During the SLB stage, air quality worsened with PM_2.5_ concentrations reaching a peak of 55.6 μg m^−3^ and an average value (42.8 ± 4.5 μg m^−3^). A total of 235,894 particles were measured during the SLB stage. Eight major sources were identified by investigating the mixing states of the total particles, including the coal burning related particles (48.1%, high-K-EC and OCEC), biomass burning particles (6.7%, high-K), vehicle emission (16.4%, EC, ECOC, K-Na-EC, and dust-EC), sea salt (9.2%), ship emission (2.7%, V) particles, dust/steeling industries (3.7%, Fe), waste incineration (6.3%, Other-HMs), and road dust (3.9%, Mg-Al).

A typical clockwise shift of wind direction was observed during the SLB stage. The daily cycle was further divided into four periods (LW, LW-SB, SB, and SB-LW) based on the prevailing wind observed at the site. Notably, the PM_2.5_ concentrations and particle numbers increased significantly during the LW-SB period when the northwest direction wind transported the pollutants to the site. In addition, the continental sourced pollutants may recirculate back to land during the transition of land wind to the sea breeze resulting in a cumulative increase in pollutants. Both individual and bulk measurements support the results, with high contributions from coal burning, biomass burning, and bulk K^+^ and NO_3_^−^. The regional transported coal burning particles significantly increased during the LW-SB period. In contrast, the ship and vehicle emissions contributed higher during the SB period, with a high sulfate concentration partially originating from the ship emission. A second small PM_2.5_ peak existed around 18:00 during the SB-LW transition period and kept general stable concentrations during the LW period. The concentrations of continental particles and nitrate returned to high levels under the influence of LW.

In this study, field evidence of continental-source pollutants backflow to land with the evolution of sea breeze was observed by the SPAMS. Coal burning, biomass burning, and waste intercalation emissions showed high contributions to the total particles during the transition period of land to the sea breeze. Although the trap of air pollution by SLB in the coastal area has been recognized and studied for several decades, this study was the first report from the perspective of high-time resolution chemical composition by single particle analysis and supplies valuable data for the numerical simulations to advance our understanding on the effect of SLB in coastal cities.

## Supplemental Information

10.7717/peerj.14116/supp-1 Supplemental Information 1 *Support information of* Ambient particle characteristics by single particle aerosol mass spectrometry at a coastal site in Hong Kong: a case study affected by the sea-land breeze. S1: Composition, possible sources, average mass spectra, and time trends of the different classes detected during the total sampling campaign (Table S1 and Figures S1 to S3) S2: Zoomed-in detail of average mass spectra of 12 major particle types classified using the Art-2a clustering algorithm during the SLB stage (Figure S4) S3: Correlations between different groups (Figure S5 to S6 and Figure S8) S4: Diurnal variations of Vehicle emission particles (EC, ECOC, dust-EC and K-Na-EC) by SPAMS (Figure S7) S5: Diurnal variation of PM_2.5_ concentration during the non-SLB period (Figure S9) S6: Mixing state of particles during the LW-SB period and the super-long range transport of air masses during C3 and C5 (Figure S10).

10.7717/peerj.14116/supp-2 Supplemental Information 2 Time series of classified particles by SPAMS and 72-h backward trajectories data.

## Additional Information and Declarations

### Data Availability

The following information was supplied regarding data availability: The raw SPAMS analysis data and 72-h backward trajectories of air mass by the HYSPLIT4 model are available in the Supplemental File.
