# Supplementary material for: Ambient particle characteristics by single particle aerosol mass spectrometry at a coastal site in Hong Kong: a case study affected by the sea-land breeze
Source: PeerJ. 2022 Oct 28;10:e14116. doi: 10.7717/peerj.14116 (PMC9620973; doi:10.7717/peerj.14116)
Supplement: Supplemental Information 1 — S1: Composition, possible sources, average mass spectra, and time trends of the different classes detected during the total sampling campaign (Table S1 and Figures S1 to S3) S2: Zoomed-in detail of average mass spectra of 12 major particle types classified using the Art-2a clustering algorithm during the SLB stage (Figure S4) S3: Correlations between different groups (Figure S5 to S6 and Figure S8) S4: Diurnal variations of Vehicle emission particles (EC, ECOC, dust-EC and K-Na-EC) by SPAMS (Figure S7) S5: Diurnal variation of PM2.5 concentration during the non-SLB period (Figure S9) S6: Mixing state of particles during the LW-SB period and the super-long range transport of air masses during C3 and C5 (Figure S10). [file peerj-10-14116-s001.docx]

***Support information of***

**Ambient particle characteristics by single particle aerosol mass spectrometry at a coastal site in Hong Kong: a case study affected by the sea-land breeze**

Nana Wang^1^, Yanjing Zhang^1^, Lei Li^2^, Houwen Wang^1^, Yunhui Zhao^1^, Guanru Wu^1^, Mei Li^2^, Zhen Zhou^2^, Xinfeng Wang^3^, Jian Zhen Yu^4, 5^, Yang Zhou^1^

^1^ College of Oceanic and Atmospheric Sciences, Ocean University of China, Qingdao, China

^2^ Institute of Atmospheric Environment Safety and Pollution Control, Jinan University, Guangdong, China

^3^ Environment Research Institute, Shandong University, Qingdao, China

^4^ Division of Environment, The Hong Kong University of Science and Technology, Clear Water Bay, Kowloon, Hong Kong China

^5^ Department of Chemistry, Hong Kong University of Science and Technology, Clear Water Bay, Kowloon, Hong Kong China

Corresponding Author:

Yang Zhou^1^

238 Songling Rd, Qingdao, Shandong Province, 266100, China

Email address: (yangzhou@ouc.edu.cn)

**This file includes**

S1: Composition, possible sources, average mass spectra, and time trends of the different classes detected during the total sampling campaign (Table S1 and Figures S1 to S3)

S2: Zoomed-in detail of average mass spectra of 12 major particle types classified using the Art-2a clustering algorithm during the SLB stage (Figure S4)

S3: Correlations between different groups (Figure S5 to S6 and Figure S8)

S4: Diurnal variations of Vehicle emission particles (EC, ECOC, dust-EC and K-Na-EC) by SPAMS (Figure S7)

S5: Diurnal variation of PM_2.5_ concentration during the non-SLB period (Figure S9)

S6: Mixing state of particles during the LW-SB period and the super-long range transport of air masses during C3 and C5 (Figure S10).

Table S1. Summary of the different classes detected during the total sampling campaign (February 22 to March 10, 2013).

|  | Groups | Hourly number | Fraction  in total | Source |
| --- | --- | --- | --- | --- |
| K-rich | high-K-EC | 756 | 34.4% | Coal burning |
|  | high-K | 376 | 17.1% | Biomass burning |
|  | K-Na-EC | 209 | 9.5% | Vehicle emission |
| Marine-sourced species | sea salt | 183 | 8.3% | Sea salt |
|  | V | 39 | 1.8% | Ship emission |
| Carbonaceous species | OCEC | 106 | 4.8% | Coal burning |
|  | EC | 88 | 4.0% | Vehicle emission |
|  | ECOC | 26 | 1.2% | Vehicle emission |
|  | dust-EC | 20 | 1.0% | Vehicle emission |
| Metal | Fe | 109 | 5.0% | Dust/steeling industries |
|  | Other-HMs | 118 | 5.4% | Waste incineration |
| Fireworks/dust | Mg-Al | 113 | 5.1% | Fireworks/dust |
| Undefined |  | 57 | 2.6% | -- |

**
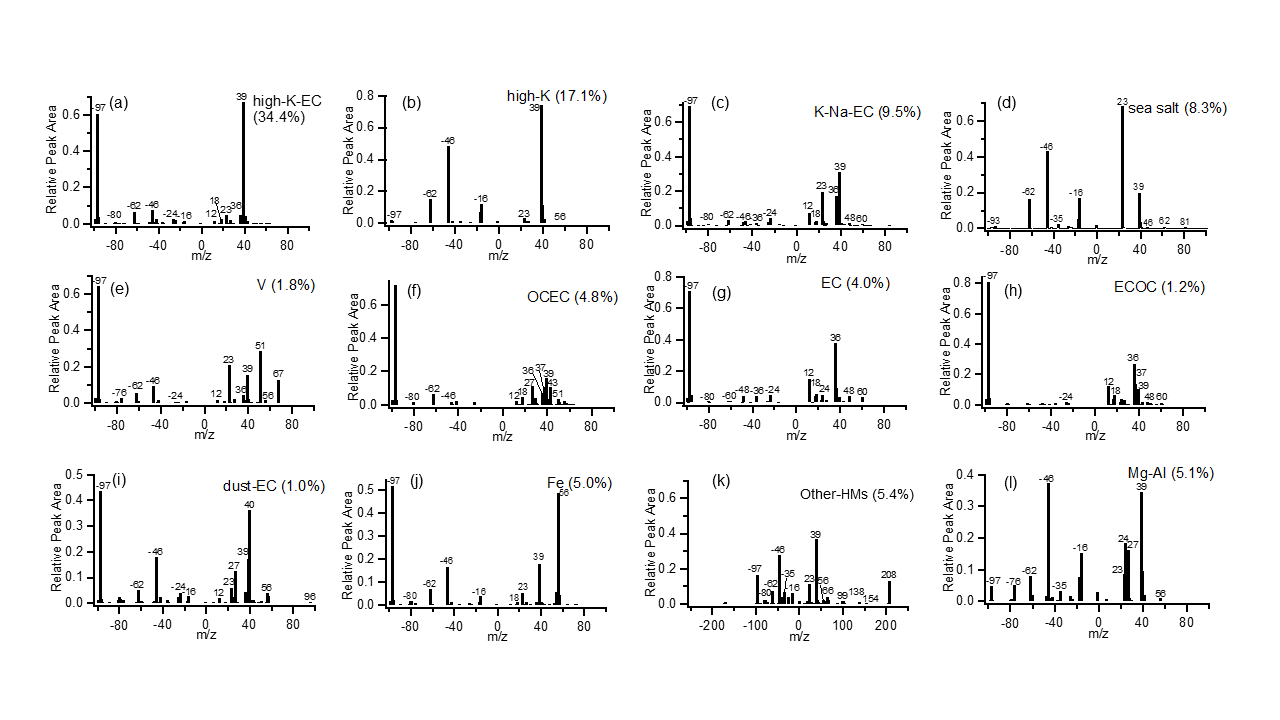
**

Figure S1. Average mass spectra of 12 major particle types classified using the Art-2a clustering algorithm during the entire observation period (February 22 to March 10, 2013).

**
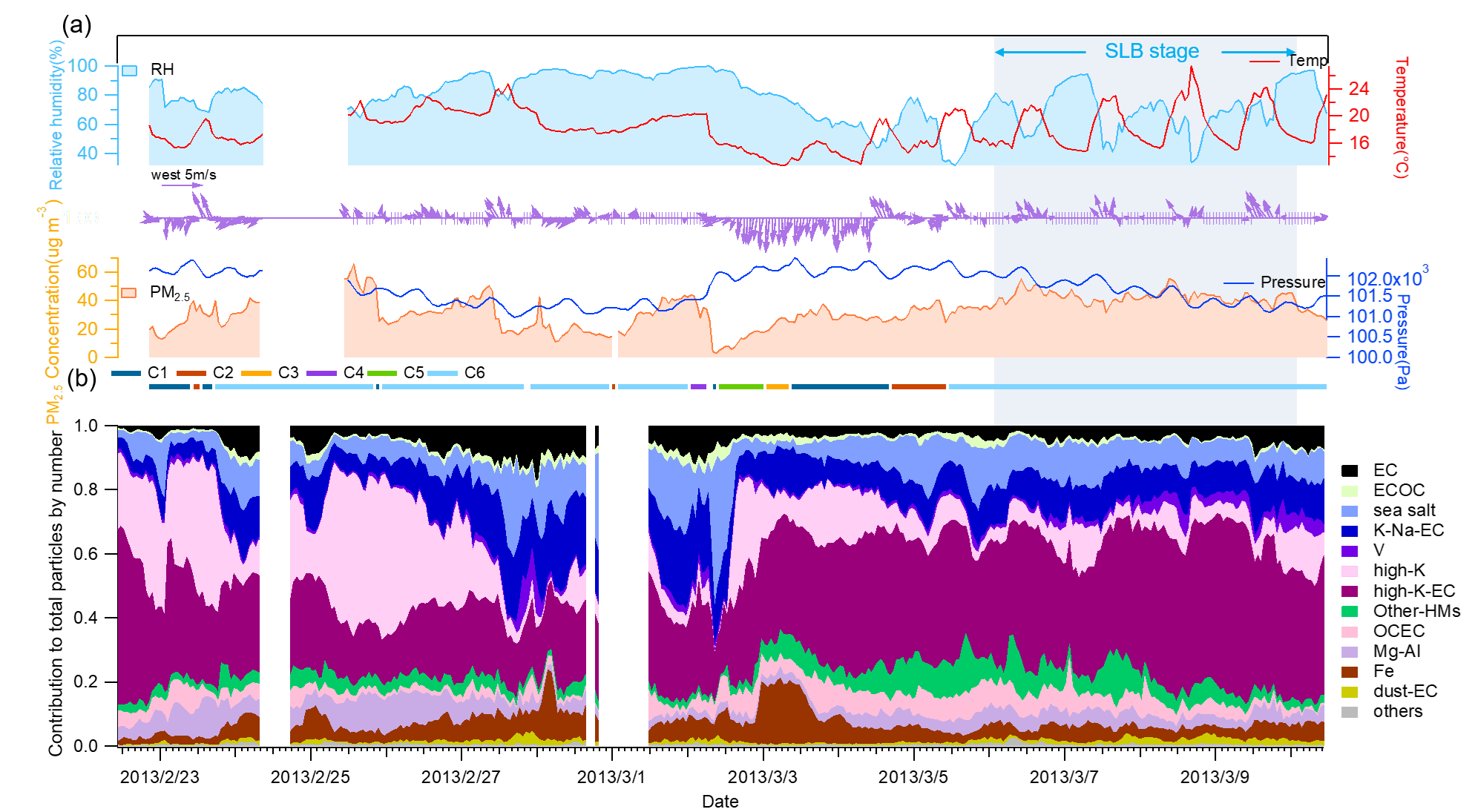
**

Figure S2. Time trends of (a) meteorological parameters, PM_2.5_ concentrations, and (b) the contribution of each group to the total particles by particle number during the entire observation period (February 22 to March 10, 2013).


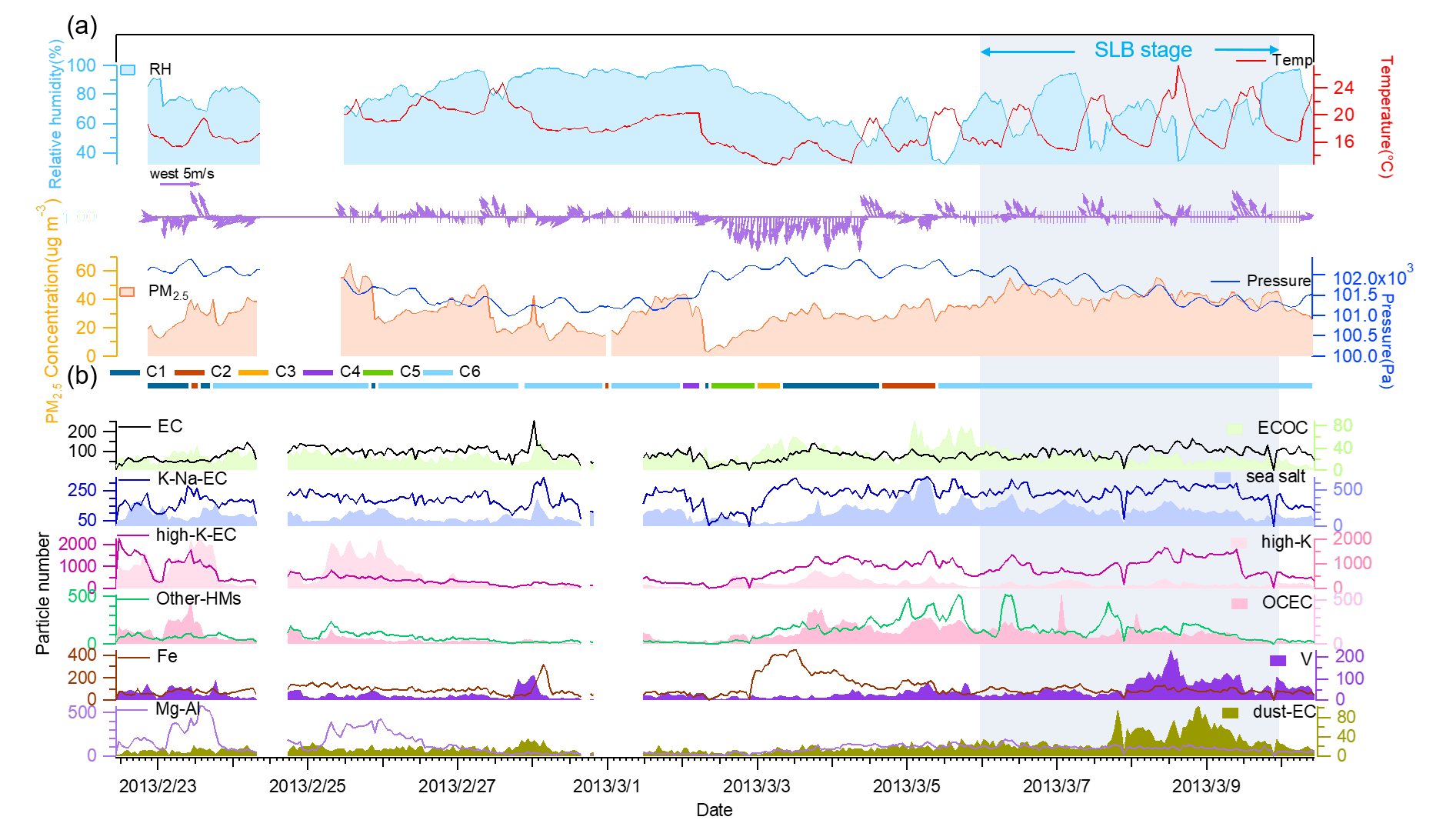


Figure S3. Time trends of (a) meteorological parameters, PM_2.5_ concentrations, and (b) the major types of classified particles, plotted by particle number during the entire observation period (February 22 to March 10, 2013).


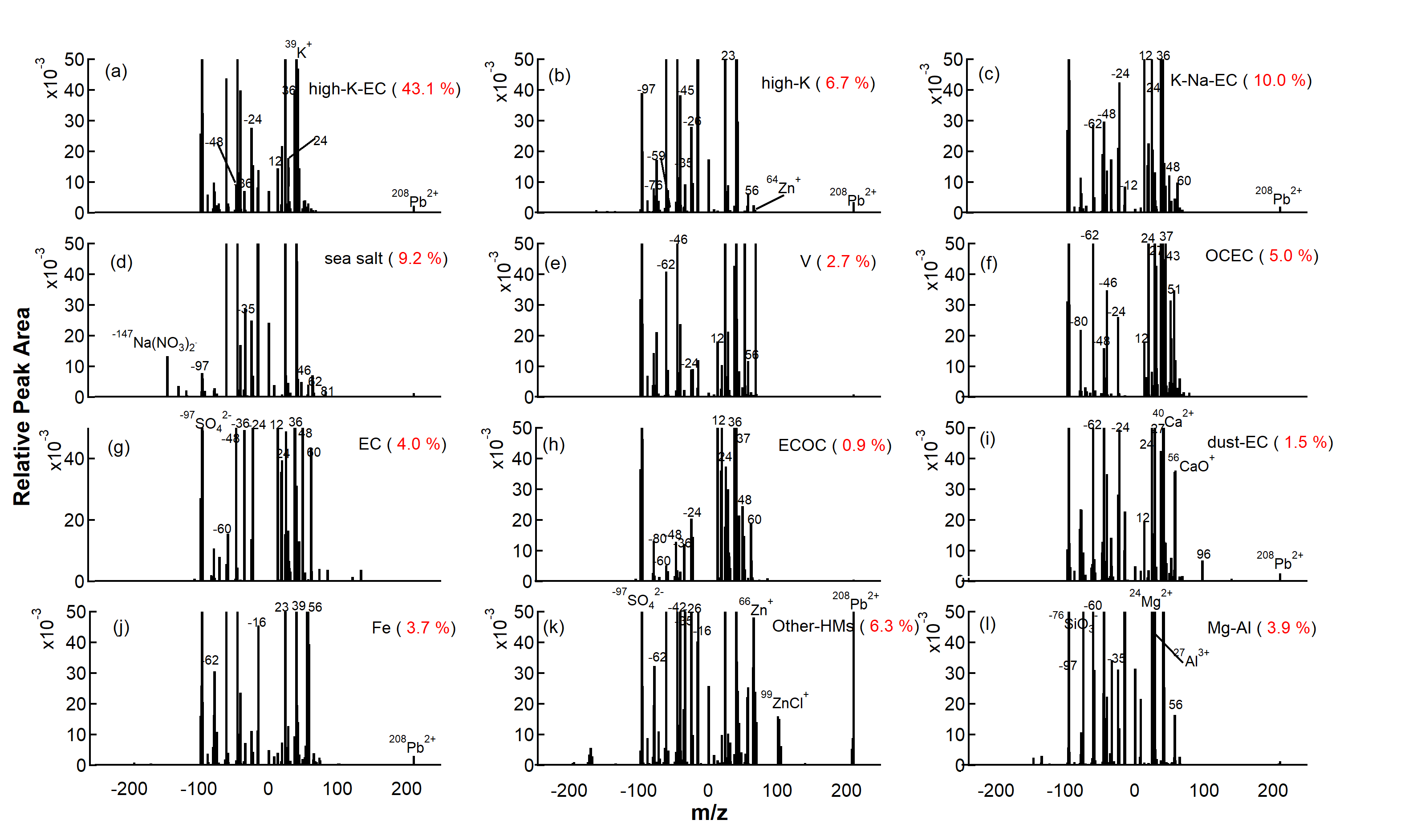


Figure S4. Zoomed-in detail of average mass spectra of 12 major particle types classified using the Art-2a clustering algorithm during the SLB stage.


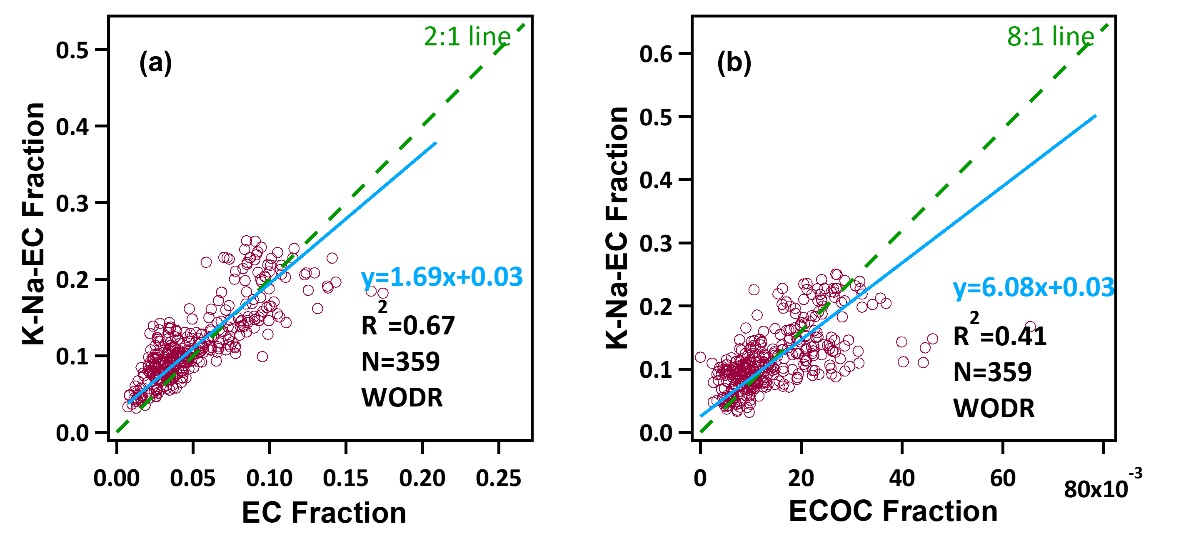


Figure S5. Scatter plots of K-Na-EC relative particles number and relative particles number of the (a) EC particles and (b) ECOC particles (Weighted ODR method was applied).


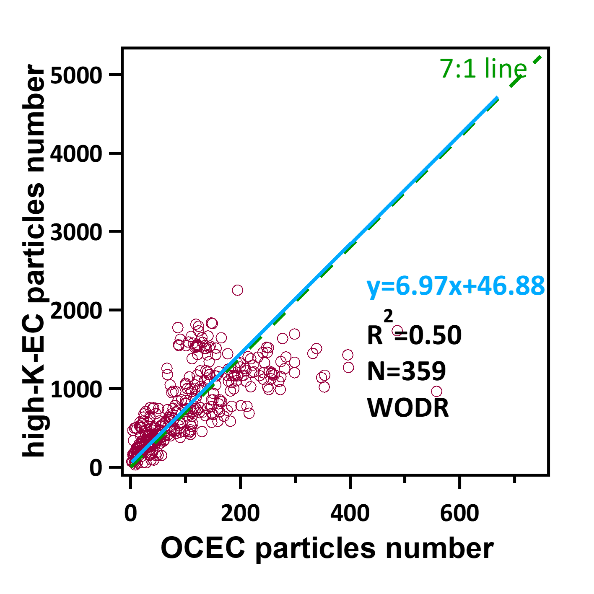


Figure S6. Scatter plot of high-K-EC particles number and OCEC particles number (Weighted ODR method was applied).


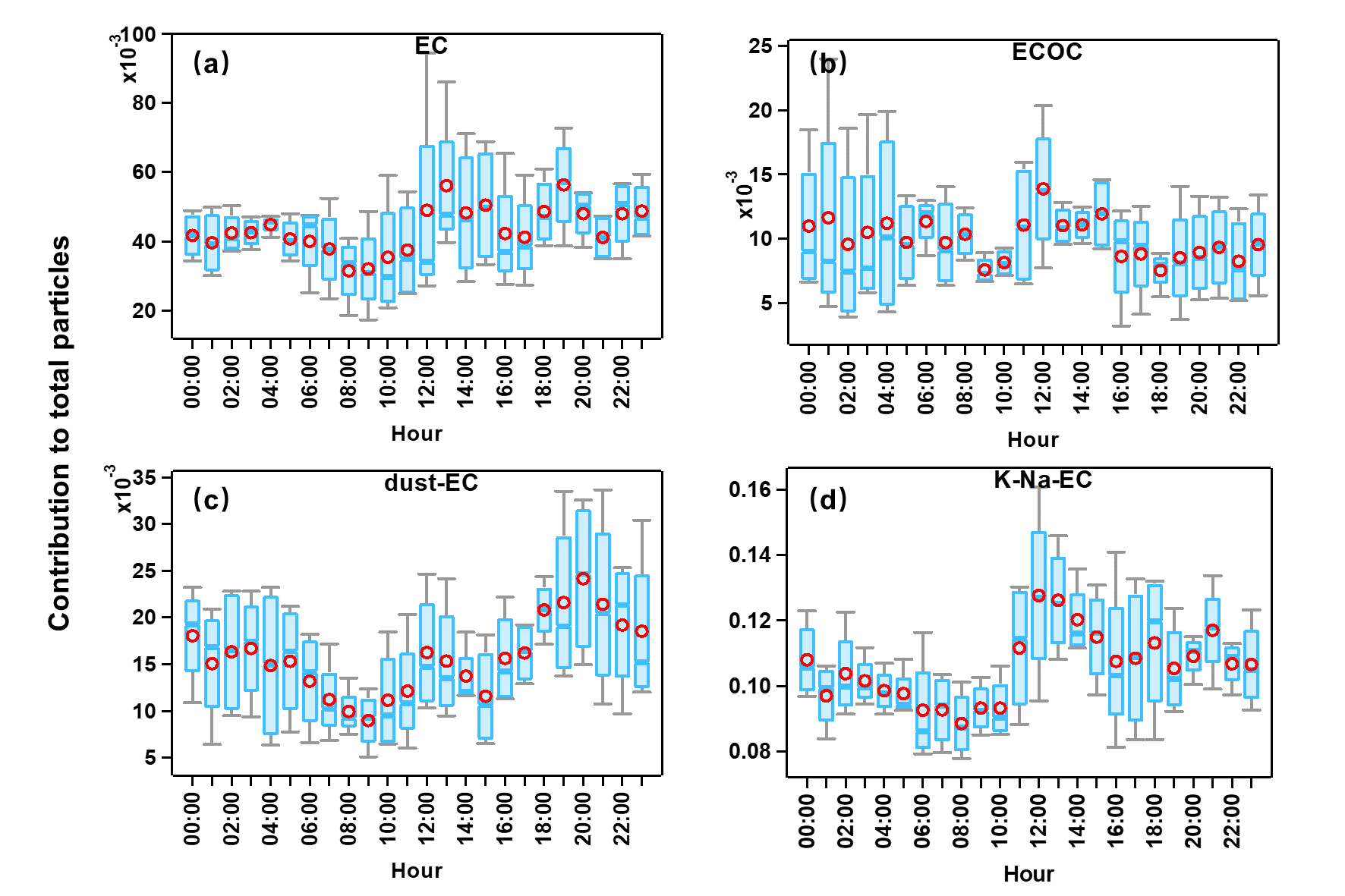


Figure S7. Diurnal variation of the contribution to the total particles by particle number of Vehicle emission particles (EC, ECOC, dust-EC and K-Na-EC) by SPAMS (Box and whisker denote the 25th and 75th percentiles, and the 5th and 95th percentiles, respectively, and the circle and line in the box represent the mean and the median).


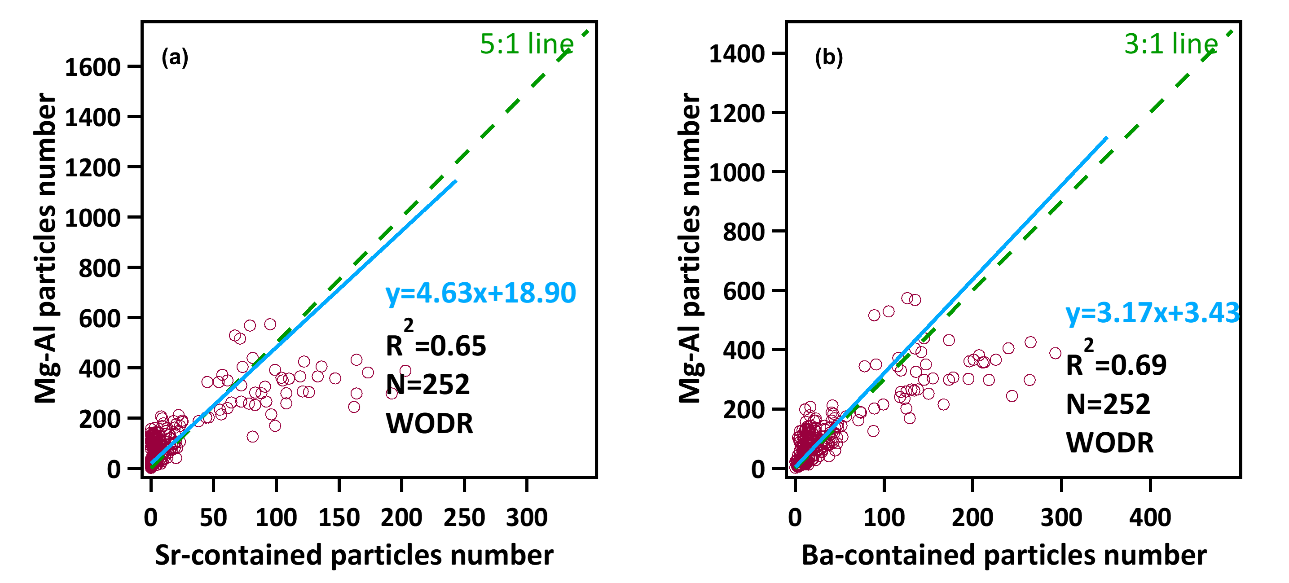


Figure S8. Scatter plots of Mg-Al particles number and particle number of the (a) Sr-containing particles and (b) Ba-containing particles during the Non-SLB stage (Weighted ODR method was applied).

**
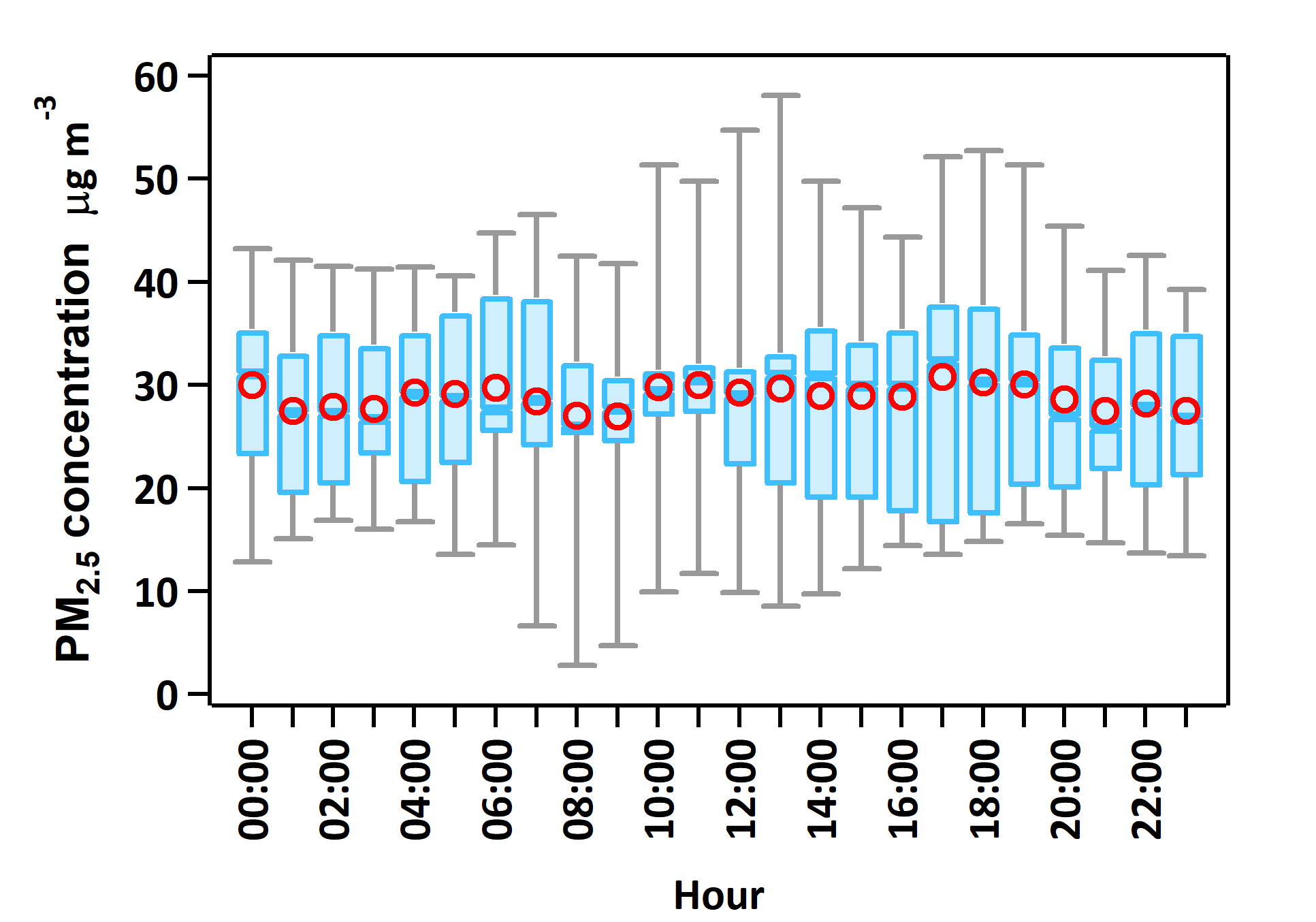
**

Figure S9. Diurnal variation of PM_2.5_ concentration during the non-SLB period.


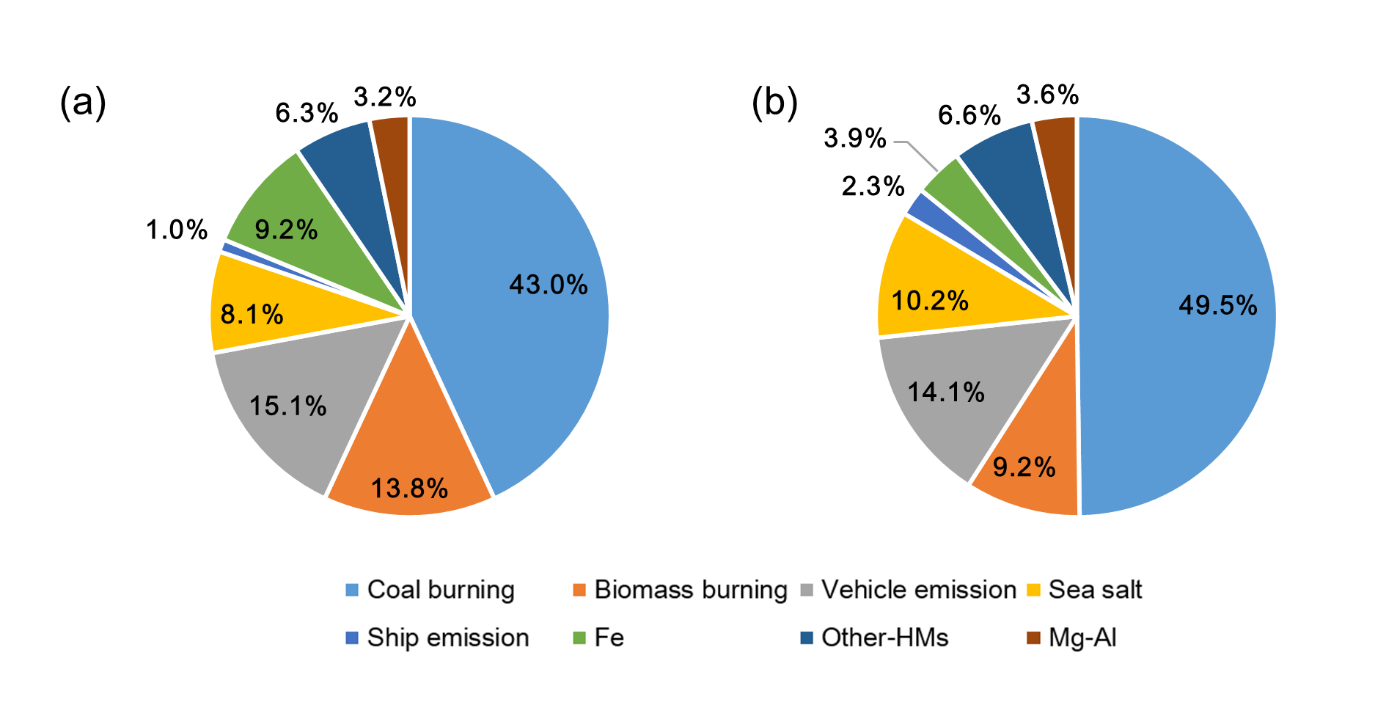


Figure S10. Contribution of particles from eight sources to the total particles by particles number during (a) the period of the super-long range transport of C3 and C5 air masses and (b) the LW-SB period during the SLB stage.
